# Anti-vinculin antibodies as a novel biomarker in Egyptian patients with systemic sclerosis

**DOI:** 10.1007/s10067-022-06301-0

**Published:** 2022-07-25

**Authors:** Noha Hosni Ibrahim, Iman Mahmoud Fawzy, Tahany Mahmoud Gouda, Rasha Abdel Hameed El Sayed, Maha Hosni Morsi, Al Shimaa Mohamed Sabry, Nashwa Ismail Hashaad

**Affiliations:** 1 Faculty of Medicine, Benha University, Benha, Al-Qalyubia Governorate, Egypt; 2 Faculty of Medicine, Tanta University, Tanta, Egypt; 3 Faculty of Applied Health Sciences Technology, Misr University for Sciences and Technology, 6Th of October City, Giza, Egypt

**Keywords:** Anti-vinculin antibodies, Interstitial pulmonary fibrosis, Pulmonary artery hypertension, Systemic sclerosis

## Abstract

**Introduction:**

Systemic sclerosis (SSc) is an autoimmune disorder that causes vasculopathy and scarring, most commonly in the lungs and skin, but it can also affect other organs. Endothelial vinculin plays a critical role in angiogenesis regulation. Therefore, vinculin overexpression in SSc may give rise to anti-vinculin antibodies, which may contribute to the development of SSc vasculopathy. The current research aims to (1) determine whether anti-vinculin autoantibodies play a significant role in the diagnosis of SSc and (2) compare anti-vinculin serum levels between two scleroderma patient populations, namely, pulmonary artery hypertension (PAH)–predominant and interstitial pulmonary fibrosis (IPF)–predominant groups.

**Methods:**

This research included 140 participants categorized into three groups: group I—patients with PAH-predominant; group II—patients with ILD-predominant; group III—the control group. Anti-vinculin antibodies were detected in serum samples collected from all participants using ELISA. All subjects underwent high-resolution computed tomography (CT), diffusing capacity for carbon monoxide, and pulmonary function tests.

**Results:**

Patients in group I (PAH-predominant group, *N* = 35) were 41.3 [± 11.4] years old, with 80% being women. Patients in group II (ILD-predominant group, *N* = 35) were 41.0 [± 11.5] years old. The SSc group showed significantly higher anti-vinculin antibody levels than the control group (*P* < 0.001). The PAH-predominant group demonstrated significantly higher anti-vinculin antibody levels and anti-vinculin positivity than the ILD-predominant group.

**Conclusion:**

Anti-vinculin antibodies in the blood appear to be diagnostic biomarkers for scleroderma. Furthermore, they shed light on some novel perspectives on the pathophysiology of specific lung fibrotic changes.

**Table:** 

**Key Points** • This study included two groups of systemic sclerosis patients (PAH-predominant group, ILD-predominant group) as well as a control group to investigate the significance of anti-vinculin antibodies in such cases. • Our results have demonstrated that anti-vinculin antibodies can play a significant role in diagnosing and monitoring systemic sclerosis disease.

## [Introduction

Systemic sclerosis (SSc) is an autoimmune disorder characterized by skin and multiple organ involvement; its underlying pathophysiologic mechanisms often include vasculopathy, autoimmunity, and fibrosis [1]. There is evidence that several stages occur prior to the onset of autoimmune disorders. Based on autoantibody findings [2] and predictive models [3], a thorough examination of these stages may lead to prophylaxis in the first instance, slowing or halting the onset of disease in the future, implying that an increase in autoantibody production contributes significantly to the clinical symptoms of many autoimmune disorders [4]. Autoantibodies in SSc have been shown to predict the clinical phenotype [5]. Vascular involvement, including PAH, has been associated with SSc. In addition, it impacts SSc pathogenesis, and endothelial cell activation may alter phenotypic presentation. Endothelial vinculin promotes angiogenesis [1]. In comparison to healthy control sera, Villano et al. found that SSc sera increased vinculin expression in endothelial cells [6] Endothelial vinculin expression and assembly must be regulated during angiogenesis [7, 8, 9].

Hence, disturbed vinculin expression may aggravate SSc’s already-defective angiogenic mechanisms [10]. In addition, anti-endothelial cell antibodies targeting antigens such as vinculin have been detected in SSc patients with and without PAH [11]. Vinculin overexpression in SSc may produce anti-vinculin antibodies, which may cause vasculopathic symptoms. According to Suliman and colleagues, PAH was linked to a significant increase in anti-vinculin antibody levels [12].

Skin and lung fibrosis in scleroderma is a maladaptive repair mechanism [1]. Fibrosis is characterized by the replacement of normal tissue structure with collagen-rich connective tissue, resulting in a rigid structure [13]. Activated vascular, epithelial, and immune cells release profibrotic cytokines, chemokines, growth factors, lipid mediators, and autoantibodies. These antibodies are present in more than 90% of patients [1, 14].

Vinculin protein is an actin-binding protein expressed in high amounts in basal bronchial cells and low amounts in alveolar cells but is not detected in ciliated cells and goblet cells [10].

Vinculin is utilized to measure cell–cell and cell–ECM (focal adhesion) adherens-type junctions, although its function is unknown [9]. Cell migration is slowed when vinculin is overexpressed, whereas it is accelerated when vinculin is downregulated. Vinculin-deficient cells are less adherent, more motile, and have fewer and smaller focal adhesions [11].

Serum biomarkers for diagnosing and tracking SSc-related lung involvement are sparse, particularly those that can identify early pulmonary interstitial fibrosis. Therefore, identifying high-risk groups for PIF remains difficult.

Scleroderma patients’ vulnerability to IPF has not been studied using serum antibodies to the vinculin protein. Serum biomarkers for diagnosing and tracking SSc-related lung involvement are sparse, especially those that can identify early pulmonary interstitial fibrosis. Still, there are concerns about identifying high-risk groups for PIF.

Scleroderma patients’ susceptibility to IPF has not been studied using serum antibodies to the vinculin protein. The current study focuses on [1] determining whether anti-vinculin autoantibodies provide insight into a diagnosis of SSc and [2] comparing serum anti-vinculin antibody levels in two SSc patient groups, namely, PAH-predominant and IPF-predominant groups.

## Materials and methods

### Study design

The current experiment is a case–control study.

#### Study participants

This study included140 subjects over the age of 18.

From December 2020 to January 2022, 70 SSc patients were admitted to Benha University Hospital’s Rheumatology and Rehabilitation Department, and a Rheumatology clinic provided serum samples. All patients fulfilled the ACR/EULAR 2013 SSc criteria.

Patients were divided into two groups based on whether PAH or IPF was predominant:

- PAH-predominant group (*N* = 35).
- IPF-predominant group (*N* = 35).

**Fig. 1:**
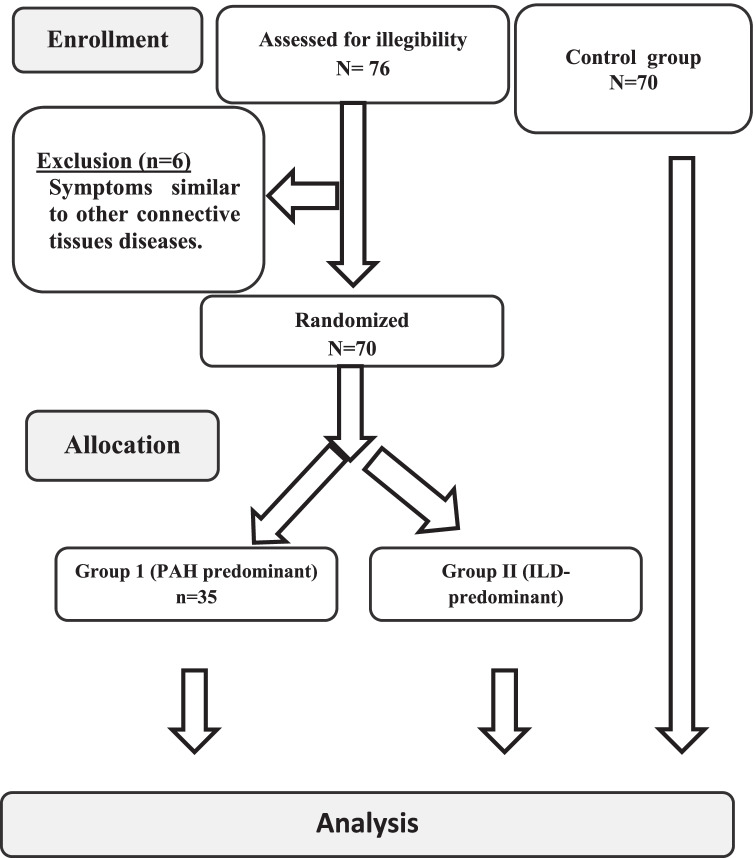
Consort chart of the study

The control group was from the general population and matched in terms of age, sex, and residence location (Fig. 1).

## Methods

### Clinical profile

We determined the clinical profile of all subjects, including demographics, past and present medical history, and respiratory symptoms such as dyspnea and cough. Patients with symptoms similar to those of other diseases of the connective tissues were excluded. In SSc patients, concurrent medications (such as vasodilators and immune suppressants) were permitted.

#### A patient's medical parameters

In SSc patients, specific clinical indicators were observed. MRSS-certified rheumatologists used clinical palpation to assess 17 body areas using the modified Rodnan Skin Score (mRSS), with each site rated from 0 to 3 for a maximum score of 51 [16]. All patients were tested for lung function and carbon monoxide diffusing capacity (DLCO) by spirometry using JAEGER CareFusion (234 GmbhLelbnizsr, Hochberg, Germany). Chest high-resolution computed tomography (HRCT) was performed for all scleroderma patients.

According to the American Thoracic Society/European Respiratory Society 2011 consensus statement, IPF was diagnosed according to the criteria for a definite diagnosis of ILD as well as chest radiography [15].

An initial echocardiogram screening was used to diagnose PAH, followed by right heart catheterization (RHC) if necessary. RHC had to have a pulmonary arterial pressure (PAP) greater than 25 and a pulmonary capillary wedge pressure (PWP) less than 15 to be diagnosed with PAH.

#### Measuring serum anti-vinculin antibody levels

A blood sample of up to 3 mL was collected from all participants for analysis.

For serum tests, whole blood samples were centrifuged for 20 min at approximately 1000 × *g*, after which the supernatant was collected and tested immediately (within 2 h).

Anti-vinculin antibody levels in serum samples from SSc patients and healthy controls were determined using enzyme-linked immunosorbent assays (ELISAs).

Cloud-Clone Corp laboratories performed ELISAs with full-length *Homo sapiens* (human) vinculin protein (Cloud-Clone Corp, Katy, USA) as antigens at a 1.2 mg/mL concentration [17].

ELISA plates were coated with recombinant vinculin. Antigens were immobilized overnight at 4 °C in borate buffered saline (Merck KGaA, Darmstadt, Germany) (Grenier Bio-One, Monroe, NC). Wells were coated with antigen or left uncoated to detect nonspecific serum binding. Wells were blocked for 1 h at room temperature with 3% BSA in PBS. Coated and uncoated wells received a 1:32 serum dilution. Following that, secondary antibodies conjugated to horseradish peroxidase were incubated (Jackson ImmunoResearch, West Grove, PA). After each step, PBS-Tween 20 was used to wash. The samples were then read using a Multiskan Microplate Photometer using a 3,39,5,59-tetramethylbenzidine substrate solution (Merck KGaA, Darmstadt, Germany) (Thermo Fisher Scientific Oy Ratastie, Finland).

Anti-vinculin levels were compared using 90-min ODs at 370 nm and studied raw OD values. The appropriate anti-vinculin cutoff was determined using a ROC curve. These OD values were obtained from both healthy and SSc ELISA individuals. The AUC was 0.947 (95% CI 0.908–0.987) at the cutoff (OD of 1.25), with sensitivity and specificity of 87.1 and 95.7, respectively. This cutoff was different from that used in previous studies [12, 18].

According to prior investigations, “consistent” results showed anti-vinculin antibodies above the OD reference range of 1.25***.*** “Inconclusive” results indicated that antibodies were below the specified range.

*Ethical consideration.*

The ethical committee for human research of Benha’s Faculty of Medicine approved the research protocol (approval reference number RC1-7–2020). All patients gave their written consent after clearly explaining the study objectives and procedures.

### Statistical analysis

Medical charts provided clinical data for statistical correlations. IBM SPSS 25 was utilized for statistical analysis. The odds ratio and 95% confidence interval for developing scleroderma were calculated using conditional logistic regression.

### Statistical power

A power analysis was performed based on the number of subjects and significant clinical effect sizes. In addition, Stats Direct software was used for sample size calculation. A two-tailed *P* value was used in all statistical analyses.

### Data analysis

The descriptive distributions of demographic and clinical parameters were analyzed, and the optimal anti-vinculin cutoff thresholds were determined using ROC curves. The AUC was calculated using the SAS LOGISTIC procedure. Subjects were categorized into two groups in light of their positivity (1.25) and negativity (1.24).

Tests based on variable type were used for comparison. Positive Abs were measured and compared to healthy controls in groups I and II.

## Results

### Subjects' demographic and clinical data

This study enrolled two SSc groups, group I (PAH-predominant, *N* = 35) and group II (ILD-predominant, *N* = 35), in addition to a control group including 70 subjects. Both SSc groups had comparable mean ages and female representation (Table 1).

**Table 1:** Evaluation of studied parameters between the PAH-predominant and ILD-predominant groups

		Group 1 (PAH predominant)	Group II (ILD-predominant)	*P* value
		*n* = 35	*n* = 35	
Age (years)	Means (SD)	41.3 (11.4)	41.0 (11.5)	0.925
Sex (%females)	*N* (%)	28 (80)	26 (74.3)	0.569
BMI	Means (SD)	26.8 (3.7)	26.8 (4.3)	0.998
PAH	Means (SD)	33.0 (4.0)	25.0 (4.0)	< *0.001*
FCV	Means (SD)	83.2 (5.5)	71.1 (5.7)	< *0.001*
DLCO	Means (SD)	82.9 (5.4)	71.7 (6.3)	< *0.001*
TLC (L)	Means (SD)	4.5 (0.9)	3.9 (1.0)	*0.004*
M Rod score	Means (SD)	38.8 (9.9)	38.4 (11.1)	0.883
Digital ulcer	*N* (%)	27 (77.1)	27 (77.1)	1
Digital gangrene	*N* (%)	13 (37.1)	11 (31.4)	0.615
Normal CT	*N* (%)	28 (80)	13 (37.1)	< *0.001*
CT nodular pattern	*N* (%)	2 (5.7)	9 (25.7)	*0.025*
CT ground-glass opacity	*N* (%)	2 (5.7)	12 (34.3)	*0.002*
CT honeycombing	*N* (%)	3 (8.6)	13 (37.1)	< *0.001*
ANA	*N* (%)	34 (97.1)	34 (97.1)	1
Anticentromere antibody	*N* (%)	25 (71.4)	16 (45.7)	*0.029*
Anti-scleroderma 70 antibody	*N* (%)	28 (80.0)	34 (97.1)	*0.024*
Anti-vinculin antibody	Median (IQR)	1.8 (1.7–1.9)	1.7 (1.2–2)	*0.014*
Anti-vinculin antibody > 1.68 (positive)	*N* (%)	35 (100)	22 (62.9)	< *0.001*

A *P* value less than 0.05 in italics is significant

#### Patients with systemic sclerosis had significantly higher levels of anti-vinculin antibodies than healthy individuals

**Fig. 2:**
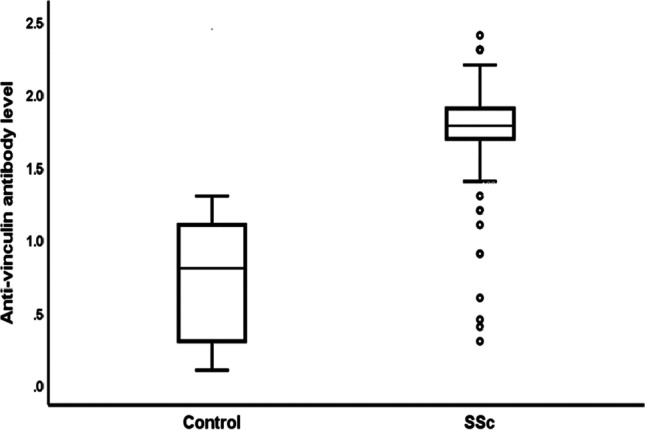
Comparing SSc (both groups) and healthy controls for anti-vinculin antibody levels

The SSc group showed significantly higher anti-vinculin antibody levels compared to the control group (*P* < 0.001) (Fig. 2).

#### Positive levels of anti-vinculin in the blood were found to be associated with PAH

The PAH-predominant group had significantly higher PAH, forced vital capacity, DLCO, total lung capacity, the proportion of normal CT, anti-centromere antibodies, lower CT nodules, and ground-glass opacities, honeycombing, and anti-scleroderma 70 antibodies than ILD-predominant group.

#### Antibody positivity rates in PAH and ILD groups were compared

PAH-predominant group had significantly higher anti-vinculin antibody levels than the ILD-predominant group (Table 2).

**Table 2:** Differences between the positive and negative groups of anti-vinculin

Group I (PAH-predominant group)				
		Anti-vinculin (negative)	Anti-vinculin (positive)	*P* value
		*n* = 13	*n* = 22	
BMI	Mean (SD)	26.4 (3.8)	27.0 (3.7)	0.653
PAH	Mean (SD)	32.9 (4.2)	33.1 (4.0)	0.932
FCV	Mean (SD)	84.5 (4.4)	82.4 (6.0)	0.276
CT: nodular opacities	*N*(%)	2 (15.4)	–	–
CT: ground-glass opacity	*N*(%)	1 (7.7)	1 (4.5)	1
Honeycombing	*N*(%)	0 (0.0)	3 (13.6)	0.279
ANA	*N*(%)	13 (100.0)	21 (95.5)	0.435
Anticentromere antibody	*N*(%)	9 (69.2)	16 (72.7)	0.825
Anti-scleroderma 70 antibody	*N*(%)	10 (76.9)	18 (81.8)	0.726
Group II (ILD-predominant group)				
		Anti-vinculin (negative)	Anti-vinculin (positive)	*P* value
		*n* = 18	*n* = 17	
BMI	Mean (SD)	27.2 (4.7)	26.4 (4.0)	0.617
PAH	Mean (SD)	25.1 (4.4)	24.9 (3.6)	0.900
FCV	Mean (SD)	70.7 (6.0)	71.6 (5.4)	0.637
CT: nodular opacities	***N***(%)	1 (5.6)	8 (47.1)	**0.007**
CT: ground-glass opacity	***N***(%)	9 (50)	3 (17.6)	**0.040**
Honeycombing	***N***(%)	2 (11.1)	11 (64.7)	**0.001**
ANA	***N***(%)	17 (94.4)	17 (100)	1
Anticentromere antibody	***N***(%)	7 (38.9)	9 (52.9)	0.404
Anti-scleroderma 70 antibody	***N***(%)	17 (94.4)	17 (100)	1

#### Establishing the cutoff for positivity

**Fig. 3:**
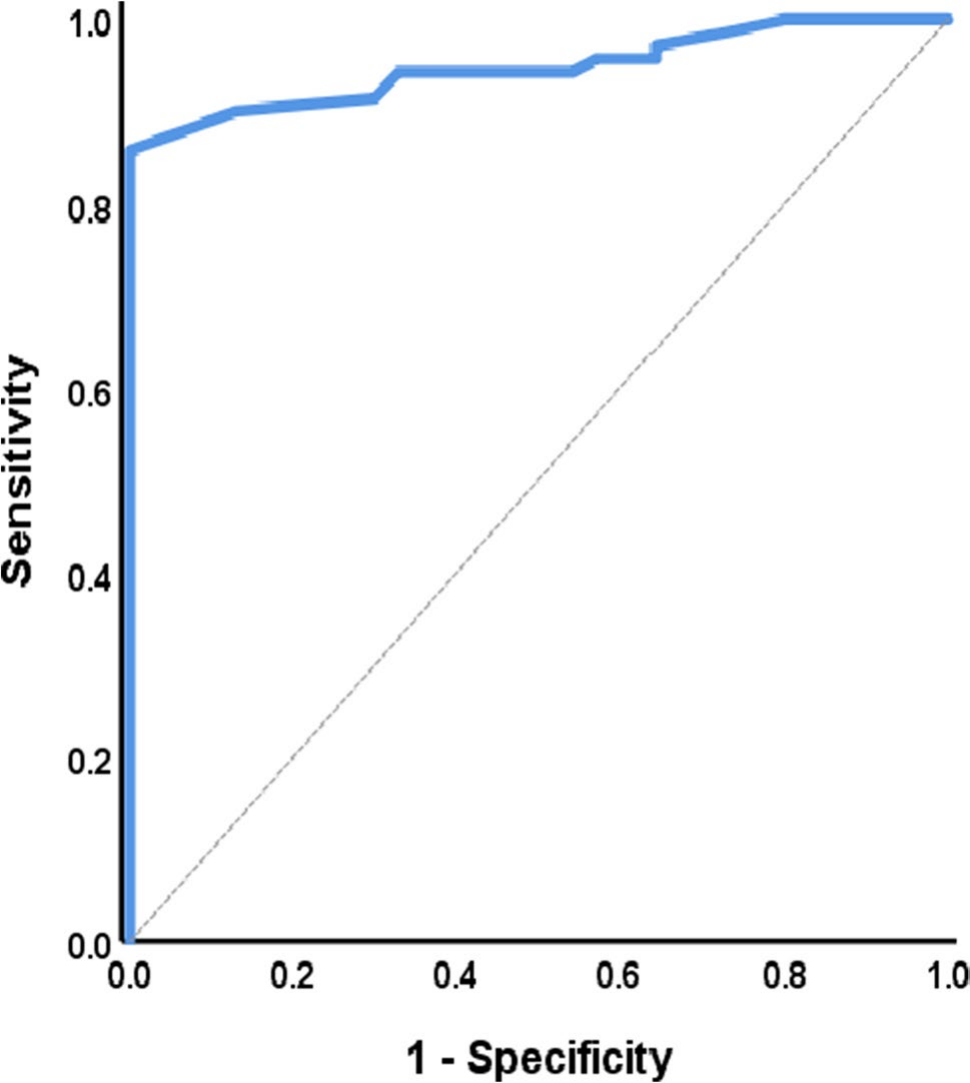
Discrimination between the SSc and control groups using the receiver operating characteristic curve (ROC)

**Fig. 4:**
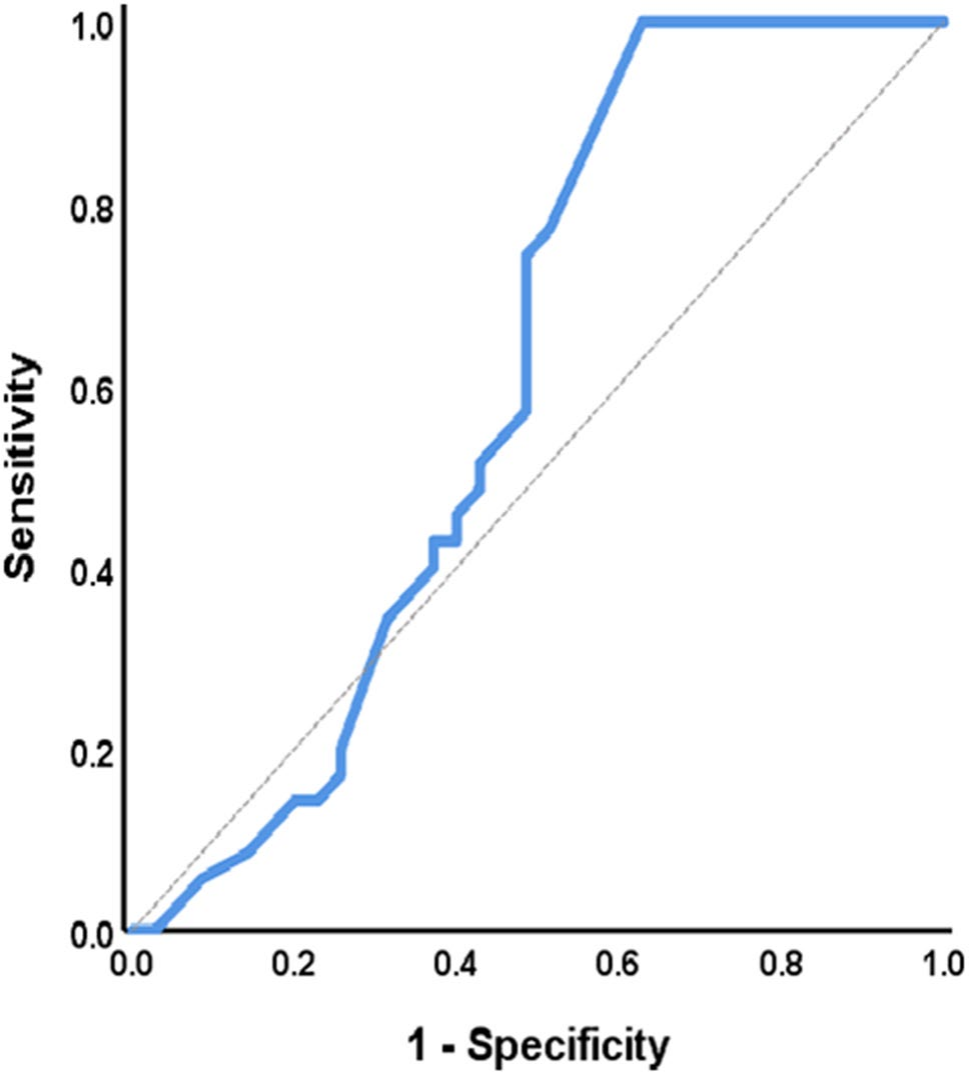
Discrimination of PAH from ILD using the receiver operating characteristic curve

ROC curves were used to determine anti-vinculin cutoff levels in SSc patients. The AUC was utilized to differentiate well between the SSC and control groups (0.947) (Fig. 3); however, it did not differentiate well between PAH and ILD (0.609) (Fig. 4). Table 3 shows the best cutoff and performance characteristics.

**Table 3:** Receiver operating characteristic curve to differentiate between studied groups

	Between control and SSc	Between PAH and ILD
AUC	0.947	0.609
95% CI	0.908–0.987	0.469–0.748
Cutoff value	1.25	1.7
Sensitivity (%)	87.1	74.3
Specificity (%)	95.7	51.4
PPV (%)	95.3	60.5
NPV (%)	88.1	66.7
Accuracy (%)	91.4	62.9

ANA, antinuclear antibody; BMI, body mass index; DLCO, diffusing capacity of the lungs for carbon monoxide; ILD, interstitial lung disease; PAH, pulmonary artery hypertension; FVC, forced vital capacity; TLC, total lung capacity.

In the ILD group, anti-vinculin positivity was significantly associated with a higher proportion of CT nodular opacity and honeycombing and a lower proportion of CT ground-glass opacity. Aside from that, no significant associations between anti-vinculin antibodies and investigated parameters were observed.

AUC, area under the receiver operating characteristic curve; ROC, receiver operating characteristic curve; CI, confidence interval; PPV, positive predictive value; NPV, negative predictive value.

## Discussion

The number of positive anti-vinculin Abs was substantially higher in SSc patients than in healthy controls in both groups (group I (PAH-predominant group) and group II (ILD-predominant group)), which is consistent with previous research findings [6] [12].

In ILD-predominant scleroderma cases, anti-vinculin positivity was associated with a higher proportion of CT nodular opacity and honeycombing and a lower proportion of CT ground-glass opacity. There were no other significant associations between anti-vinculin Abs and the parameters studied. Vinculin, a cytoskeletal protein, is expressed in the lungs. This protein promotes fibrosis by participating in cell–cell and cell–ECM adhesion [19]. Vinculin dysregulation may aggravate pulmonary fibrosis in SSc patients. We hypothesized that vinculin overexpression in SSc increased anti-vinculin Abs, contributing to interstitial lung fibrosis.

To our knowledge, this is the first study to investigate the use of anti-vinculin antibodies in scleroderma patients with ILD considering that our search of PubMed for studies was published in January 2022 using the following query ("Pulmonary Fibrosis"[Mesh]) AND "Scleroderma, Systemic"[Mesh]). Furthermore, "Vinculin"[Mesh] yielded no results. Hence, more research is needed to evaluate if anti-vinculin antibodies can be utilized to diagnose pulmonary fibrosis in SSc patients.

Our study showed that the PAH group showed significantly higher anti-vinculin antibody levels and positivity than the ILD group. This conclusion is consistent with Suliman et al.’s [12] suggestion that anti-vinculin was only related to PAH, highlighting the need to examine a broader sample to demonstrate this potential correlation [12].

Before vinculin antibody measurements can be used, disease-specific normal and abnormal values must be established. Based on the values of the patient and control groups, we discovered that 1.25 was the optimal cutoff for diagnosing SSc using patient populations and healthy controls, which is inconsistent with the findings of Pimentel et al. [20]. However, we assessed anti-vinculin antibodies in IBS patients, not scleroderma patients, and our optimum cutoff for SSc diagnosis is incompatible with Suliman et al. (OD of 1.68) [6] [12]. The variance in cutoff values can be attributed to sample size, ethnicity, location, test chemicals, and measurement instruments.

## Implications for future research

Our research has a wide range of implications. First, our findings suggest that in SSc, vinculin protein may play a role in the pathogenesis of the vascular and fibrotic changes detected in the ECM and cell migration.

Based on the results of previous studies implicating vinculin as a potential target of anti-endothelial antibodies in SSc, it appears that vinculin may play an essential role in SSc pathophysiology when disrupted by an autoimmune process.

The current study’s strengths include the following: the appropriate selection of participants, the strict diagnosis of scleroderma following the ACR/EULAR 2013 SSc requirements, the inclusion of a control group for comparison, and the exclusion of patients with symptoms overlapping those of other connective tissue diseases. To our knowledge, ILD in scleroderma patients has never before been studied with anti-vinculin antibodies. Furthermore, this study could differentiate between PAH and IPF groups, two of the most common pathologies involved in SSc and have been linked to significant mortality.

However, it should be noted that the current study has some limitations. First, our sample may be representative of only the Egyptian population. There were two distinct SSc groups from one hospital. Consequently, our findings cannot be generalized.

Second, there is no agreement on the cutoff for normal and abnormal values in the diagnosis of SSc. More research in different geographic areas will be required to determine region-specific cutoffs for background antibody levels required to differentiate true disease from healthy individuals. In addition, further investigation is required to evaluate the difference in cutoff antibody levels between PAH and ILD subjects and the lower specificity for the cutoff differentiating PAH from ILD. Further research with a larger sample size will be needed to verify whether anti-vinculin cutoff levels can be used for the diagnosis of scleroderma and to differentiate between PAH and ILD.

The best cutoff value for a diagnostic test can be determined by considering the prevalence of the disease of interest in the study population and the costs (not just financial) of false-negative (FN) and false positive (FP) results [21].

## Conclusion

Anti-vinculin antibodies may serve as biomarkers for scleroderma, allowing scleroderma to be an inclusion diagnosis rather than an exclusion diagnosis, and may aid in the targeting investigations in those who tested negative, given that not all scleroderma subjects test positive for this biomarker. In addition, anti-vinculin antibodies may provide new insights into the pathophysiology of some lung fibrotic changes, such as CT nodular opacity and honeycombing in ILD associated with scleroderma. The subsequent stage of our research involves investigating the potential mechanisms by which this biomarker contributes to the development of some lung fibrotic changes in subjects with ILD associated with scleroderma.

## Data Availability

The datasets used and/or analyzed during the current study are available from the corresponding author on reasonable request.

## Declarations

### Patient involvement

Patients were not involved in the design, conduct, reporting, and dissemination plans of this article.

## Abbreviations

SSc: Systemic sclerosis
PAH: Pulmonary artery hypertension
ILD: Interstitial lung disease
FN: False negative
FP: False positive
ECM: Extracellular matrix
mRSS: Modified Rodnan Skin Score
IPF: Interstitial pulmonary fibrosis
RHC: Right heart catheterization
PAP: Pulmonary arterial pressure
PWP: Pulmonary capillary wedge pressure
DLCO: Diffusion lung capacity of carbon monoxide
HRCT: High-resolution computed tomography
